# An AI-based algorithm for analyzing physical activity and health-related fitness in youth

**DOI:** 10.1038/s41598-026-35514-5

**Published:** 2026-01-13

**Authors:** Mei Lv, Jing Wang, Yunqiang Yang, Jinghua Zhong, Jianlou Yang, Chen Dong

**Affiliations:** 1 School of Physical Education Department, Shandong Women’s University, Jinan, 250300 China; 2 School of Computer Engineering, Weifang University, Weifang, 216000 China; 3 Jinan Jingwu Road Primary School, PE Groups, Jinan, 250001 China; 4 Linyi Education and Sports Bureau, Linyi, 251500 China; 5 School of Sport Management, Shandong Sport University, Jinan, 250102 China

**Keywords:** Fitness management and assessment, Physical health, Exercise science, Artificial intelligence, Computational biology and bioinformatics, Engineering, Health care, Mathematics and computing

## Abstract

In recent years, with the country’s emphasis on national fitness, the health status of primary and secondary school students has become the focus of social attention. As one of the important means to measure students’ physical fitness, physical examination results are closely related to students’ physical fitness. However, there are some problems in the traditional physical examination management, such as subjective influence, complicated manual calculation, and difficulty in retaining and making full use of data. Based on the physical fitness test data of primary schools in the past five years from 2018 to 2022, this study aims to apply machine learning and deep learning methods to deeply analyze and mine data information, provide automatic classification methods and accurate performance prediction models, and then expand to provide students with personalized training suggestions to assist teachers in making reasonable teaching plans and other applications. The first research method is the classification method based on BP neural network, which realizes automatic comprehensive grade classification and achieves 98.448% classification performance, and explores students’ physical health and grade classification. The second research method is the performance prediction model based on CNN-LSTM neural network, which combines CNN feature matrix and LSTM continuous time series information to provide more accurate performance prediction for various physical test items, and provides a new method for the management and evaluation of physical test results of primary and secondary school students through data analysis and prediction model. These methods not only solve the problems of traditional evaluation methods, but also provide scientific guidance for schools and promote the healthy development of students and the optimization of physical education.

## Introduction

As the main members of the future society, the health status and physical quality of primary and secondary school students are directly related to the health and stability of the country and society^1,2^. Physical fitness test is one of the important ways to test students’ physical fitness in schools, and the performance of physical examination is closely related to physical fitness^3,4^. The performance can represent the ability of physical fitness from different dimensions, for example, the 50-m dash represents the explosive ability, the sitting body flexion represents the body flexibility ability, the vital capacity test represents the cardiopulmonary ability, and skipping rope for one minute can show endurance and physical coordination at the same time. The purpose of physical examination for primary and secondary school students is to encourage college students to learn exercise and improve their physical health. At the same time, physical fitness test can cultivate students’ awareness of physical exercise from primary schools, and the test results can assist physical education curriculum design.

While physical fitness tests in schools are instrumental in shaping tailored physical education programs and informing health-related policies, there exists a notable gap in the traditional management and analysis of these assessments. The physical fitness testing process faces inherent challenges. Firstly, the detection and analysis phases are characterized by a significant investment of time and labor. Secondly, the presence of subjective influences and manual recording errors, such as data loss, misrecording, and scoring discrepancies, poses a threat to the reliability of physical test results. Thirdly, the analysis of physical measurement data introduces variability into the results, and the lack of standardized evaluation criteria leads to inconsistent comprehensive score calculation standards over the years. Lastly, the existing management scheme is deficient in leveraging data for forecasting and planning, as it predominantly focuses on basic data preservation and analysis in the traditional approach to handling physical examination results. The traditional management of physical examination results only realizes the preservation of performance data and simple analysis, and lacks the mining of information. The complexities surrounding the evaluation process reveal inherent challenges that impede the efficacy and accuracy of physical fitness assessments.

As society transitions into an era marked by advancements in artificial intelligence (AI), there emerges an opportune moment to address these inherent challenges^5,6^. This study aims to bridge the gap by proposing innovative machine learning and deep learning methodologies, seeking to automate and enhance the accuracy of physical fitness assessments in schools. With the development of artificial intelligence, the existing research results use deep learning to analyze and predict athletes’ performance and academic analysis and early warning^7^. Deep neural network has a powerful ability to find hidden relationships, patterns and trends from a large number of data and provide support for decision-making. Therefore, based on the above physical measurement management and the shortcomings of existing methods, how to establish a high-precision physical fitness test performance prediction model deserves great attention^8^.

The purpose of this study is to use machine learning and deep learning to solve the problems existing in the evaluation of school physical examination results. In view of the problems existing in the traditional manual evaluation method, this paper intends to propose a classification method based on BP neural network, which can accurately classify the physical test scores in an automatic way and provide scientific basis for students’ physical health evaluation^9,10^. This model can fully explore the complex relationships in data and realize the automatic and objective evaluation process. On this basis, this paper designs a performance prediction model based on CNN-LSTM to predict the project performance for many years^11^ and assist teachers to make more reasonable teaching plans. This study focuses on the rationality of performance evaluation, prediction efficiency and standardization of calculation standards, reducing the workload of manual calculation, and exploring the practical significance of artificial intelligence in the field of sports. The subsequent sections will delve into the specific methodologies employed and their implications for transforming the landscape of physical fitness evaluation.

Several pioneering studies have explored the intersection of AI and physical fitness assessment, seeking to revolutionize the conventional paradigms. The two mainstream methods for modeling and predicting learning performance mainly include machine learning (ML)-based models^12^ and deep learning (DL)-based models^13^. Various machine learning (ML) methodologies have been employed in educational research, moving beyond traditional statistical techniques such as multiple regression^14^ and stepwise regression^15^. These statistical methods, reliant on assumptions of data normality, often falter when faced with the complexities of real-world data^16^. In response to the limitations of statistical approaches, ML techniques have gained prominence for their robust handling of large datasets. Noteworthy, ML-based algorithms utilized in predicting academic performance encompass, among others, artificial neural network (ANN)^17^ and support vector machine (SVM). Ma et al.^18^ utilized a tree-based classification model named ID3 to extract physical fitness patterns hidden in the data. Zhou et al.^19^ utilized ensemble learning method on physical health data, by combining different classification algorithms they improved the results. Insights gleaned from previous ML models reveal that diverse factors, including demographics, engagement levels, and ongoing assessment scores, play pivotal roles in influencing future performance in fitness test.

Recent studies have showcased the effectiveness of deep learning models in accurately predicting individual performance outcomes across diverse domains, spanning sports and academics. Wang et al.^20^ pioneered the integration of big data analytics and data mining, devising a model capable of robustly assessing the quality of physical education teaching in college settings. Another study by Wang et al.^21^ delved into the exploration of the correlation between athletic ability and physical health through innovative data mining approaches. Fang et al.^22^ proposed a model based on BP networks, successfully enhancing the management proficiency of school sports departments and elevating the overall quality of physical education. Furthermore, the synergy of fuzzy analysis and neural networks led to the development of comprehensive algorithms and fuzzy neural network algorithms. These advancements collectively embrace a data-driven paradigm, offering a predictive lens into physical fitness achievements.

Although various DL-based algorithms have been extensively researched in the field of academic performance prediction, the Long Short-Term Memory (LSTM) network^23^, as a distinctive deep learning model, has been relatively overlooked in the context of predicting physical fitness scores over multiple years (e.g., six years of elementary school). Compared to other deep learning models, LSTM exhibits unique strengths in handling time-series data and sequential tasks, allowing it to better capture long-term dependencies within the data. In the prediction of physical fitness scores, effectively capturing these intricate temporal dependencies is of paramount importance. Therefore, in this paper, we innovatively incorporate LSTM into the physical fitness score prediction model, combining convolutional neural networks (CNNs) and long short-term memory networks (LSTMs), which has demonstrated significant potential in analyzing complex datasets.

This study extensively explores the application of machine learning and deep learning methods in enhancing the assessment of physical health. We establish a comprehensive evaluation index system for primary and secondary school students, conduct quantitative analyses of each index, and employ the analytic hierarchy process to determine the weights of these indices. Simultaneously, we develop a duration-based ranking of physical fitness scores and a predictive model for individual future scores, providing valuable insights for the design of the next phase of student physical education training and curriculum planning related to physical health.

## Materials and methods

The purpose of this study is to make full use of artificial intelligence technology to assist physical fitness monitoring, and to use multiple methods to model, analyze and predict physical fitness data, which not only provides scientific guidance for practice, but also deeply analyzes the correlation between multiple characteristics. With the continuous development of big data platforms and tools, it provides a more intelligent choice for scientific analysis of physical measurement data. In this study, the analysis of physical measurement data is completed by data mining technology, and the grade classification and performance prediction of physical measurement data are realized by deep neural network. Combining the concepts of big data, data analysis and artificial intelligence, this paper organically combines the three to provide a general scheme for sports-related work.

**Fig. 1:**
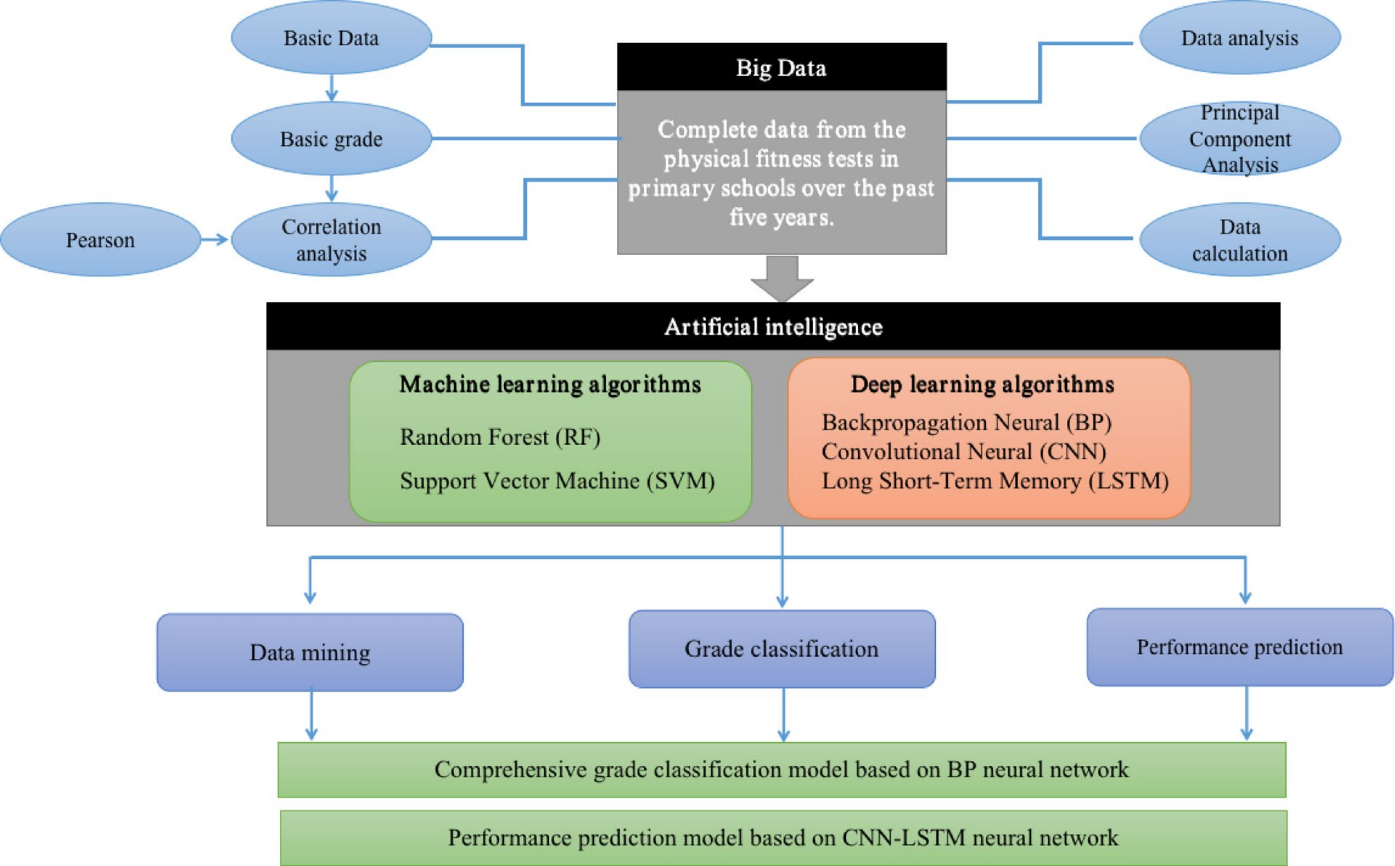
Overall research program of the paper.

The overall technical route is shown in Fig. 1. Firstly, in the analysis stage of physical measurement data, this paper makes an exploratory analysis of the comprehensive measurement data of students from grade one to grade six. First of all, analyze the physical health status of students in different grades and the correlation between various indicators. Then, these data are standardized, and on this basis, principal component analysis is carried out to extract more representative information. Secondly, BP neural network algorithm is designed to realize the classification of comprehensive grades. In this paper, the characteristics of physical measurement data are introduced into the model for training, and the physical fitness test model is applied to other years to predict the comprehensive grades, so as to observe the difference between the prediction results of the model and the actual teachers’ manual calculation results. Thirdly, realizing the performance prediction model based on CNN-LSTM neural network. The CNN layer transforms the features into one-hot vectors and forms a matrix. Then, the LSTM model is used to extract the features of the year series to predict the performance of various physical tests, which provides a practical method for optimizing the healthy development of students and physical education.

### Physical measurement data analysis

Taking primary and secondary school students’ physical health test results in recent five years from 2018 to 2022 as the research sample, the data includes students’ personal basic information and project physical measurement data, in which the basic data includes height, weight and BMI, and the project physical measurement data includes vital capacity, 50-m running, sitting forward, skipping rope for one minute and sit-ups. Records include equipment test results, individual test scores, individual test grades, additional scores, and comprehensive scores calculated manually. There are 13,706 samples, and some data are shown in Table 1. Sit-ups are not assessed for Primary 1 and Primary 2 students, as this test is not required at these grade levels. Before responding to any questions, all participants were given an informed consent form. This form detailed the study’s purpose, the survey’s content, and how the data would be used. Participants were required to sign the informed consent form before continuing with the survey, and they could withdraw from the study whenever they wished. This study protocol received full ethical approval from the Ethics Review Committee of the School of Physical Education Department, Shandong Women’s University. All methods were performed in accordance with the relevant guidelines and regulations.

**Table 1:** Example table of partial data for physical test items.

Year	Grade name	Height	Weight	BMI	Lung capacity	50 run	Seated forward bends	Rope skipping	Sit-ups	Total grade
2022	Primary 1	131.5	38.2	22.10	931	12.4	7.5	53		Pass
2022	Primary 1	134.5	29.5	16.30	851	11.2	5.4	140		Outstanding
2022	Primary 2	127.0	31.5	19.50	1372	12.0	13.5	131		Good
2022	Primary 2	138.5	26.7	13.90	941	10.9	9.0	167		Outstanding
2022	Primary 3	145.0	46.5	22.10	1559	9.8	8.0	139	30	Good
2022	Primary 3	136.5	39.0	20.90	1300	10.7	0	131	20	Pass
2022	Primary 4	140.0	31.9	16.30	1640	9.6	20.0	180	57	Outstanding
2022	Primary 4	145.0	50.3	23.90	1333	10.9	10.0	159	39	Good
2022	Primary 5	143.0	31.7	15.50	2530	10.3	10.5	154	32	Good
2022	Primary5	153.5	40.2	17.10	1957	11.1	11.4	144	31	Pass
2022	Primary 6	155.0	32.1	13.40	1479	9.1	15.2	156	50	Good
2022	Primary 6	155.5	38.9	16.10	2530	8.8	21.0	175	44	Outstanding

In this paper, before establishing the model of grade classification and performance prediction, the data are cleaned and pre-processed, including missing values and abnormal values, to ensure that the data are compared on the same scale, and the normalization is limited to [− 1,1], and the formula is as follows.

1 $$x_{{{\mathrm{norm}}}} { = }\frac{{x_{i}^{A} {\text{ - min}}_{A} }}{{{\mathrm{max}}_{A} {\text{ - min}}_{A} }}$$

Among them,${\text{ x}}_{{\mathrm{i}}}^{{\mathrm{A}}}$ represents the ith value of column A, ${\mathrm{min}}_{{\mathrm{A}}}$ and ${\mathrm{max}}_{{\mathrm{A}}}$ represent the maximum and minimum values of the column respectively. Then standardize the data, the formula is as follows,

2 $$x_{std} = \frac{{x_{i}^{A} - \mu_{A} }}{{\sigma_{A} }}$$

Among them, $\mu_{A}$ and $\sigma_{A}$ represent the mean and standard deviation of the scores in column A, respectively.

Then, this paper uses principal component analysis and correlation analysis to decompose multiple attributes, get the importance between measurement items and comprehensive scores, and calculate the correlation between each item. Taking the data in 2018 as an example, this paper uses Pearson correlation coefficient to calculate the normalized and standardized data, and the relevant formulas are as follows.

3 $$r = \frac{{\sum\nolimits_{i = 1}^{n} {\left( {x_{i} - \mathop{x}\limits^{\leftharpoonup} } \right)} \left( {y_{i} - \mathop{y}\limits^{\leftharpoonup} } \right)}}{{\sqrt {\sum\nolimits_{i = 1}^{n} {\left( {x_{i} - \mathop{x}\limits^{\leftharpoonup} } \right)^{2} } } \sqrt {\sum\nolimits_{i = 1}^{n} {\left( {y_{i} - \mathop{y}\limits^{\leftharpoonup} } \right)^{2} } } }}$$

where ${\mathrm{x}}_{\mathrm{i}}$ and ${\mathrm{y}}_{\mathrm{i}}$ represent two attributes, $\mathop{x}\limits^{\leftharpoonup}$ and $\mathop{y}\limits^{\leftharpoonup}$ represent their corresponding mean values. After calculation, the correlation coefficient between the comprehensive score and each item is shown in the following table.

As can be seen from Table 2, the ranking of the relationship between each item and comprehensive performance is skipping rope, sitting forward, 50-m running, sit-ups and vital capacity, in which positive and negative are positive and negative.

**Table 2:** Correlation coefficients between composite scores and individual items.

	Height	Weight	Lung capacity	50 m run	Seated forward bends	Rope skipping	Sit-ups
Total grade	–	–	− 0.0276	− 0.1991	0.3822	− 0.6933	0.1521
Rank	–	–	5	3	2	1	4

The results of correlation coefficient are shown in Table 3. There is a strong positive or negative correlation between the measured items, for example, there is a positive correlation between height, weight and vital capacity, and there is a strong negative correlation between height and one-minute skipping. Because the data correlation is too strong, it is not easy to transfer the model to the data of other years. Therefore, this paper uses the principal component analysis method to convert the original data into the input data needed by the model to avoid the disadvantages caused by the coupling of features. Before the experiments, we explicitly clarify that PCA was applied to the standardized physiological feature set prior to model training. The number of retained components was determined based on the cumulative explained variance criterion. Specifically, we retained the first six principal components, which together account for 95.3% of the total variance in the original feature space. This choice represents a balance between dimensionality reduction and information preservation. By retaining over 95% of the variance, the PCA-transformed input preserves the dominant structure of key physical fitness indicators, while effectively reducing feature redundancy and mitigating multicollinearity caused by strong correlations among raw physiological variables.

**Table 3:** Correlation of individual attributes in 2018.

	Height	Weight	Lung capacity	50 m run	Seated forward bends	Rope skipping	Sit-ups
Height	1.0000	0.6201	0.6656	− 0.5607	− 0.2451	0.6991	0.1305
Weight	0.6201	1.0000	0.5422	− 0.2771	− 0.1877	0.5401	0.3332
Lung capacity	0.6656	0.5422	1.0000	− 0.5377	− 0.1552	0.6388	0.0799
50 m run	− 0.5607	− 0.2771	− 0.5377	1.0000	0.1799	0.1911	0.6301
Seated forward bends	− 0.2451	− 0.1877	− 0.1552	0.1799	1.0000	− 0.2109	0.6619
Rope skipping	0.6991	0.5401	0.6388	0.1911	− 0.2109	1.0000	− 0.2481
Sit-ups	0.1305	0.3332	0.0799	0.6301	0.6619	− 0.2481	1.0000

### Comprehensive grade classification model based on BP neural network

**Fig. 2:**
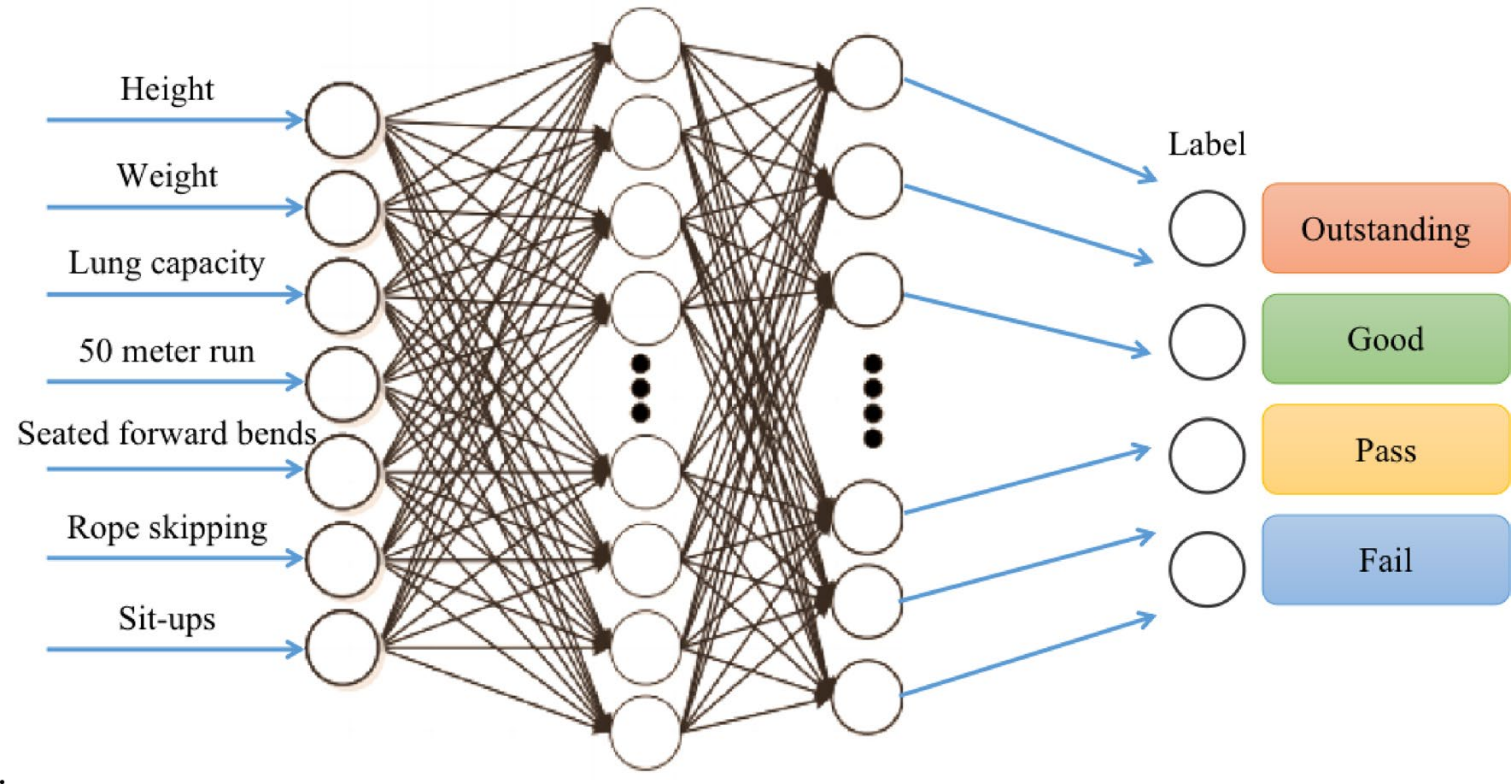
Overall structure of BP neural network.

In this study, BP neural network model is used to realize the comprehensive grade classification of physical test results, which provides an efficient and accurate method for students’ physical health evaluation. The data provided in this paper will be divided into four grades: failing, passing, good and outstanding. The model includes data preprocessing, neural network construction, training process, parameter setting and other basic processes. BP neural network is composed of input layer, hidden layer and output layer (Fig. 2).

As shown in Fig. 2, the input layer data are physical data such as height and grade, weight and grade, BMI and grade, vital capacity and grade, 50-m running and grade, sitting forward and grade, skipping rope for one minute and grade, and the output layer is divided into four categories, corresponding to outstanding, good, collective and failing grades respectively. The specific realization formula is,

4 $$\begin{aligned} z_{{\text{hidden }}} { = } & W_{{{\text{input\_hidden }}}} \cdot x + b_{{\text{hidden }}} \\ a_{{\text{hidden }}} { = } & \frac{{1}}{{{1 + }e^{{{ - }z{\text{ hidden }}}} }} \\ \end{aligned}$$

Then, each neuron uses the Softmax activation function, and the calculation formula is,

5 $$\begin{aligned} z_{{\text{hidden }}} { = } & W_{{{\text{input\_hidden }}}} \cdot x + b_{{\text{hidden }}} \\ a_{{\text{hidden }}} { = } & \frac{{1}}{{{1 + }e^{{{ - }z{\text{ hidden }}}} }} \\ \end{aligned}$$

where x is the input feature vector, W_input_hidden_ is the weight matrix from the input layer to the hidden layer, b_hidden_ is the bias of the hidden layer, W_input_hidden_ is the weight matrix from the hidden layer to the output layer, and b_output_ is the bias of the output layer.

In the training process, this paper uses the cross entropy loss function as the objective function, and the calculation formula is,

6 $$L = - \sum\nolimits_{i = 1}^{{n_{output } }} {y_{true,i} \log \left( {a_{output ,i} } \right)}$$

where y_true_, i is the one-hot coding form of the actual comprehensive level.

In this paper, the error of output layer and hidden layer is calculated by back propagation algorithm, and the weight and offset are updated by gradient descent method. The physical measurement data from 2018 to 2022 are all trained and tested by the classification model. The data sets are randomly divided into training set 10,964, verification set 1371 and test set 1371, with a ratio of 8:1:1.

### Performance prediction model based on CNN-LSTM neural network

**Fig. 3:**
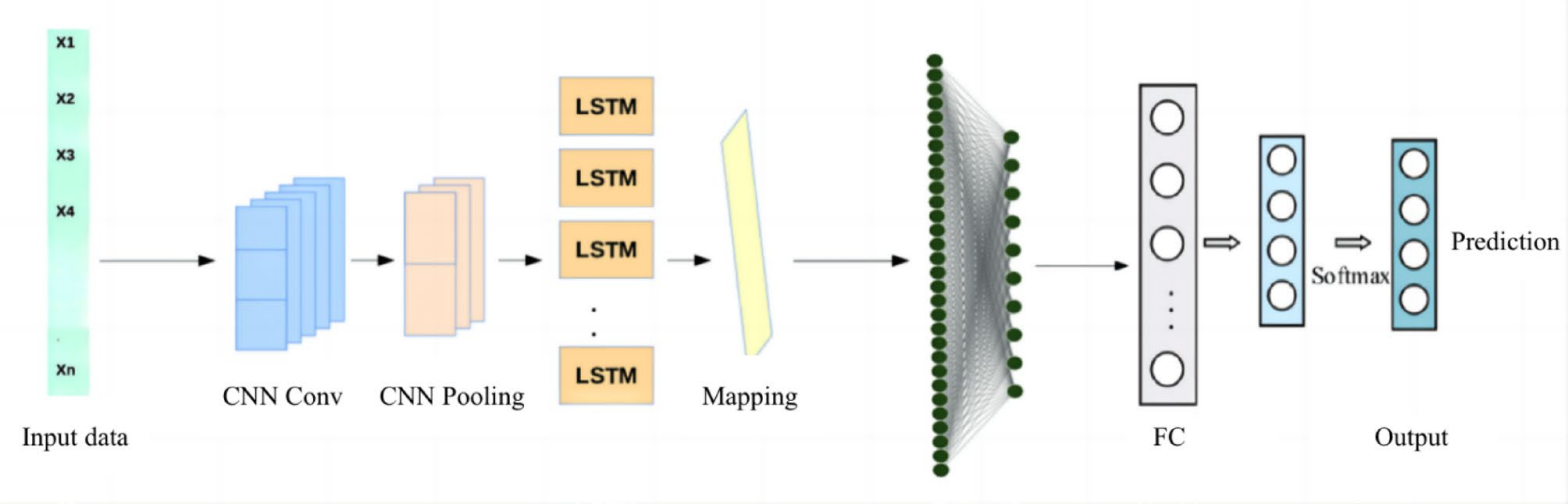
Framework of CNN-LSTM neural network performance prediction model.

Artificial intelligence technology can realize the classification of grades and learn the nonlinear mapping relationship between multiple projects by using BP model, but it can’t model the prediction of future achievements. Based on the temporal characteristics of the data in recent five years, this paper further introduces CNN-LSTM to establish a long-term learning and forecasting model. Based on the students’ physical test data and corresponding scores, the physical test scores of each student are sorted in chronological order. Combined with the characteristics of time series in recent five years, a neural network model is established and trained to predict the results of the project. The overall framework of the model is shown in the following figure (Fig. 3).

The model consists of several CNN layers, LSTM layers, attention mechanism and full connection layers. Among them, CNN layer includes convolution layer and pooling layer, which are used to extract the characteristics of each physical measurement item, while LSTM is used to extract the time series characteristics. Attention layer is to give weight to the features learned by CNN and LSTM, and to provide guarantee for accurately predicting students’ grades. The fully connected layer compresses the output features of the LSTM layer to the final features, and realizes the prediction of students’ grades through the Softmax classifier. The hidden layer of CNN includes more convolution layers, pooling and fully connected layers.

In this paper, after several rounds of automatic training by using CNN network, the gap between the predicted results of each project and the measured results is minimized, and the function of accurately predicting the physical fitness monitoring results is realized. The model takes the data from 2018 to 2021 as the training set and the data from 2022 as the test set, which is convenient for the prediction of the results in 2023. The input layer in CNN’s concrete implementation process is characterized by ${\text{X = }}\left\{ {{\mathrm{x}}_{{{\mathrm{i1}}}} {\mathrm{,x}}_{{{\mathrm{i2}}}} {,} \ldots {\mathrm{,x}}_{{{\mathrm{in}}}} } \right\}$, and the conv formula,

7 $$y_{ij}^{I} = \sigma \left( {\sum_{m = 1}^{M} w_{m}^{I} x_{i = m - 1}^{I - 1} + b_{j}^{I} } \right)$$

where ${\mathrm{y}}_{{{\mathrm{ij}}}}^{{\mathrm{I}}}$ is calculated from ${\mathrm{x}}_{{{\mathrm{ij}}}}$,${\upsigma }$ and ${\mathrm{m}}$ are activation function and index value respectively, ${\text{w }}$ is weight matrix, and ${\text{b }}$ is bias term. Then, the maximum pooling method learns the characteristics of importance. The formula is as follows,

8 $$p_{ij} ^{I} = \max_{r \in R} \left( {y_{i \times T,j}^{I - 1} } \right)$$

where T and R are step size and size, respectively.

**Fig. 4:**
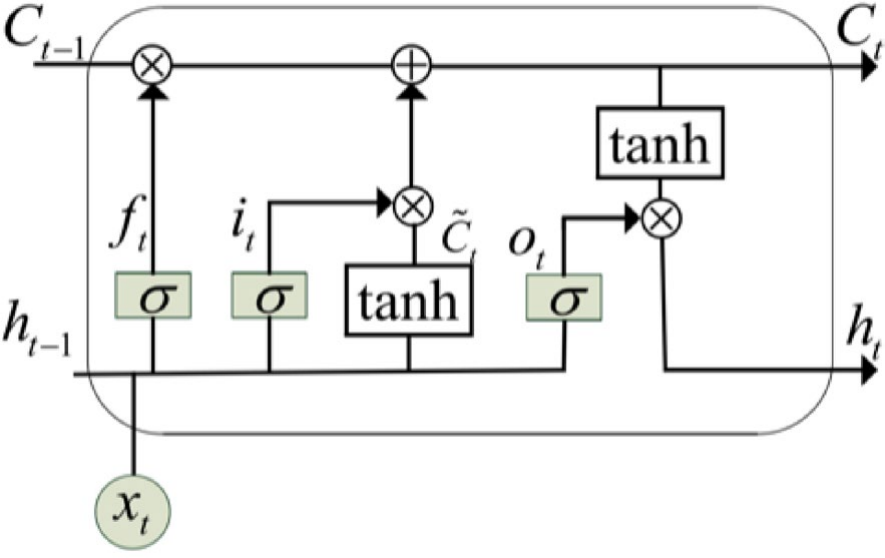
LSTM gate structure.

LSTM, on the other hand, keeps continuous information for many years after CNN. It changes the state of the network layer and updates the time sequence to predict students’ grades through operations such as input gate, forgetting gate and output gate. The structure of each LSTM is shown in Fig. 4.

The gate controller includes three types: input gate, forgetting gate and output gate. The structural unit expressions of each gate are respectively,

9 $$\begin{gathered} i_{t} = \sigma \left( {W_{1}^{i} \cdot x_{t} + W_{h}^{i} \cdot h_{i - 1} + b_{i} } \right) \hfill \\ f_{t} = \sigma \left( {W_{1}^{f} \cdot x_{t} + W_{h}^{f} \cdot h_{i - 1} + b_{f} } \right) \hfill \\ \tilde{C}_{t} = \tanh \left( {W_{1}^{C} \cdot x_{t} + W_{h}^{C} \cdot h_{t - 1} + b_{C} } \right) \hfill \\ \end{gathered}$$

The expression of each neuron state update is,

10 $$C_{t} = f_{t} *C_{t - 1} + i_{t} *\tilde{C}_{t}$$

where $b_{i} ,b_{f} ,b_{o}$ and ${\mathrm{b}}_{{\mathrm{c}}}$ are offset terms. $W_{1} ^{i} ,W_{1} ^{f} ,W_{1} ^{ \circ }$ and ${\mathrm{W}}_{{1}} { }^{{\mathrm{c}}}$ are three gate structures and output weights, respectively. Finally, the output layer of CNN-LSTM model adopts fully connected layer, and the formula is as follows,

11 $$y_{ij} ^{\prime} = \sigma \left( {w_{ij}^{I - 1} v_{t} + b_{i}^{I - 1} } \right)$$

where ${\mathrm{w}}_{\mathrm{ij}}^{{\mathrm{I}}-{1}}$ represents the weight of the ${\mathrm{i}}$ node of the ${\mathrm{I}}-{1}$ layer and the ${\mathrm{j}}$ node of the ${\mathrm{I}}$ layer. The model captures time series and spatial characteristics, and improves the accuracy and interpretability of prediction.

Then, we have explicitly the formulation of the attention layer applied to the LSTM outputs. Let $\mathrm{H}\mathrm{=}\left\{{\mathrm{h}}_{1},{\mathrm{h}}_{2},{...,\mathrm{h}}_{\mathrm{T}}\right\}$ denote the sequence of hidden states produced by the LSTM, where ${\mathrm{h}}_{\mathrm{T}}$ represents the hidden state at time step *t*, and T is the length of the input sequence. First, an intermediate attention score et is computed for each time step as:

12 $${\mathrm{e}}_{{\mathrm{t}}} {\text{ = tanh(w}}_{{\mathrm{a}}} {\mathrm{h}}_{{\mathrm{t}}} {\text{ + b}}_{{\mathrm{a}}} {)}$$

where ${\mathrm{b}}_{{\mathrm{a}}}$ are trainable parameters. The normalized attention weight ${\upalpha }_{{\mathrm{t}}}$ is then obtained using the softmax function. Using these attention weights, a context vector c is computed as the weighted sum of the LSTM hidden states:

13 $${\text{attention = }}\mathop \sum \limits_{{{\mathrm{t}} = 1}}^{{\mathrm{n}}} \frac{{{\mathrm{exp}}\left( {{\mathrm{e}}_{{\mathrm{t}}} } \right)}}{{\mathop \sum \nolimits_{{{\mathrm{k}} = 1}}^{{\mathrm{T}}} {\mathrm{exp}}\left( {{\mathrm{e}}_{{\mathrm{k}}} } \right)}}{\mathrm{h}}_{{\mathrm{t}}}$$

Finally, the context vector c is fed into the fully connected layer to generate the final prediction. This attention mechanism enables the model to dynamically focus on the most informative historical time steps, thereby improving both prediction accuracy and interpretability.

## Results

### BP grade classification model

In this paper, the real test data are used for experiments, and the data set is divided into training set and test set. Using BP neural network to classify the physical test results comprehensively can fully explore the complex relationship between data and realize the automatic and objective evaluation process. In this paper, the physical fitness test data from 2018 to 2022 are selected to establish the model, and 80% of the training set is used to optimize and adjust the model for several rounds. The evaluation indexes of the experiment are Accuracy, Recall, Precision and F1-Score, and the calculation formulas are respectively.

14 $$\begin{aligned} Accuracy = & \frac{TP + TN}{{TP + TN + FP + FN}}, \\ {\mathrm{Re}} call = & \frac{TP}{{TP + FN}}, \\ \Pr ecision = & \frac{TN}{{TN + FP}}. \\ F1 - Score = & \frac{{2 \times \Pr ecision \times {\mathrm{Re}} call}}{{\Pr ecision + {\mathrm{Re}} call}} \\ \end{aligned}$$

where TP represents the number of positive classes predicted as positive classes, TN represents the number of negative classes predicted as negative classes, FN represents the number of positive classes predicted as negative classes, and FP represents the number of negative classes predicted as positive classes. In this paper, the effect of physical measurement classification task is deeply discussed. By comparing the performance of different models in accuracy, precision and recall, the experimental results are shown in the following table.

In the classification task of physical test grade, SVM model shows a certain classification ability, and its accuracy in training and test data reaches 88.120% and 86.921% respectively. However, the accuracy and recall of SVM model are slightly lower, which are 85.006% and 87.273% respectively. This may mean that the model has certain classification bias in some cases. In contrast, BP neural network model performs well in the classification task of physical measurement grade, and has achieved outstanding results in accuracy, precision and recall. In the training data, the accuracy of BP neural network model is as high as 98.448%, and it remains stable in the test data, reaching 97.128%. In addition, the accuracy and recall of the model are 97.671% and 98.427%, respectively, which shows that the model has outstanding classification performance in all aspects. As shown in Table 4, the BP neural network achieves an F1-score of 0.9805, while the SVM baseline obtains an F1-score of 0.8613. The consistently high precision and recall values indicate that the proposed BP model maintains balanced classification performance across different fitness grade categories, rather than being biased toward dominant classes. (Table 5).

**Table 4:** Comparative test table for grade classification.

Model	Accuracy/train	Accuracy/test	Precision	Recall	F1-score
SVM	0.8812	0.86921	0.85006	0.87273	0.8613
BP	0.98448	0.97128	0.97671	0.98427	0.9805

**Table 5:** Model experimental setup.

Model parameters	Value
Convolution kernel size	1 * 2
Pooling size	1 * 3
Filtering	128
Dropout	0.3
Batch size	16
Epoch	20

### CNN-LSTM prediction model

For this model, this chapter uses PyTorch to realize CNN-LSTM model based on attention mechanism, uses logarithmic loss function and gradient descent method to train and update parameters constantly, sets the batch number to 16, and trains the model for 20 cycles. The specific experimental settings are shown in the following table.

In addition, this paper provides the experimental results, which show that the average accuracy rate, average accuracy rate and average recall rate of student performance prediction of different projects tested by different models are shown in Table 6.

**Table 6:** Comparison of achievement prediction effects.

Model	Accuracy/train	Accuracy/test	Precision	Recall	F1-score
CNN	0.87067	0.86216	0.84633	0.85345	0.8499
LSTM	0.84448	0.82233	0.83756	0.82274	0.8301
CNN-LSTM	0.9284	0.90221	0.89122	0.9027	0.8969

As can be seen from Table 6, the accuracy of CNN model in physical test performance prediction training reached 87.067%, and the test data remained at a high level of 86.216%. This shows that CNN model can effectively capture the spatial characteristics of physical measurement data. However, there is still room for improvement in accuracy and recall. Secondly, the LSTM model also has certain advantages in the prediction of physical test results. It achieved 84.448% accuracy in training data, and 82.233% in test data also showed good generalization ability. However, LSTM is slightly insufficient in comprehensive performance.

The accuracy, precision and recall of CNN-LSTM model proposed in this paper reach 92.840%, 89.122% and 90.270% respectively, which highlights its outstanding performance in the task of predicting physical test results. CNN-LSTM model effectively integrates spatial and temporal information through multi-layer feature extraction and time series modeling, and provides highly accurate prediction ability for individual physical test score prediction. As shown in Table 6, the proposed CNN-LSTM model achieves the highest F1-score (0.8969), indicating a better balance between precision and recall compared with standalone CNN and LSTM models. This demonstrates the effectiveness of combining spatial feature extraction with temporal dependency modeling for longitudinal achievement prediction.

**Fig. 5:**
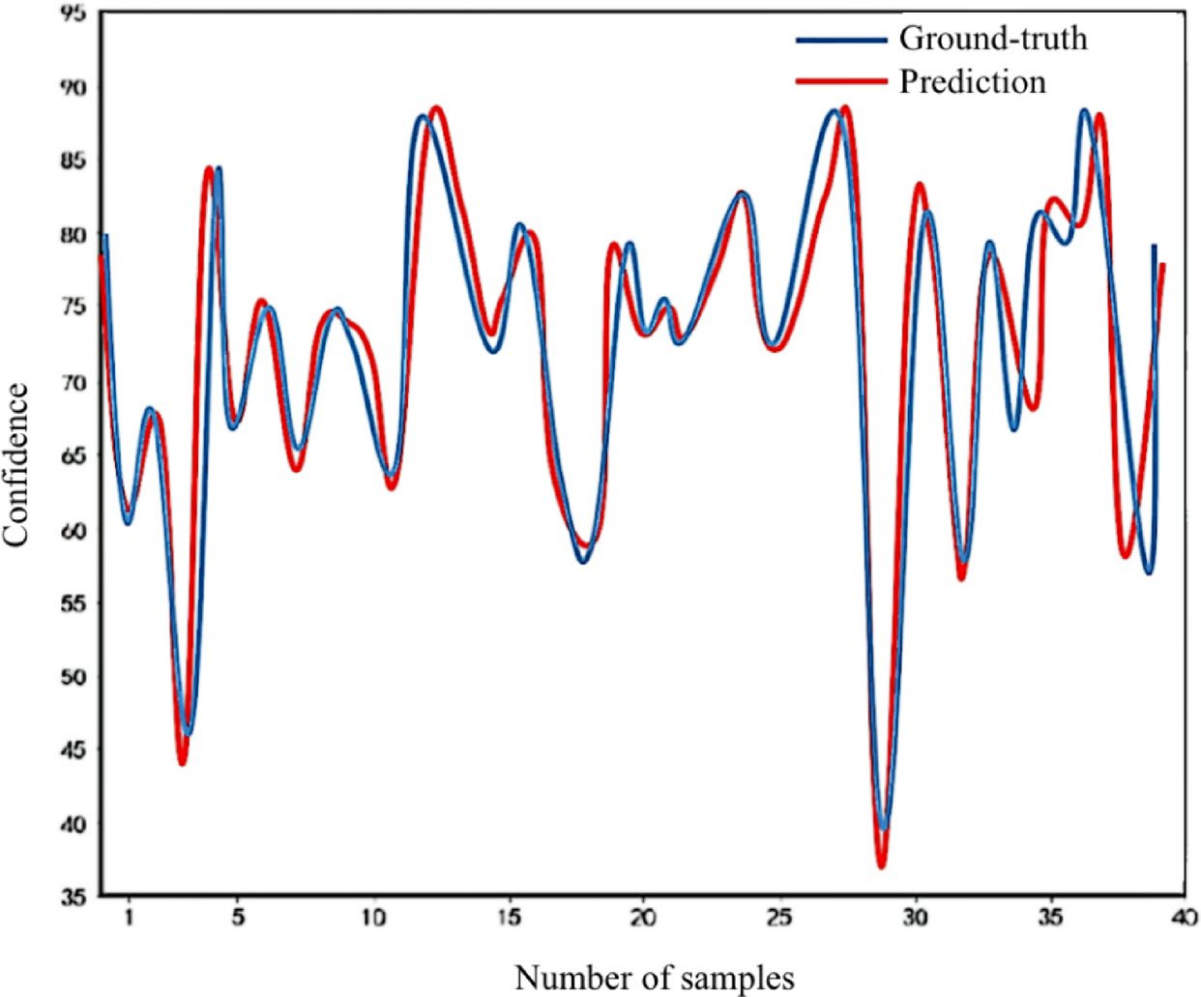
Comparison of actual and predicted physical test scores for 40 students. The Y-axis represents the measured physical test scores for each student, while the X-axis indicates the sample index.

At the same time, in order to further intuitively show the effect of the performance prediction of the physical test, this paper randomly selects 40 samples from the test samples to compare the real value with the predicted value, and displays them in the form of line charts (Fig. 5). The Y-axis represents the physical test scores, and the X-axis corresponds to the sample index. As shown, the predicted values closely follow the actual scores, with minimal deviations, indicating that the CNN-LSTM model effectively captures both spatial correlations among test items and temporal dependencies across years. This visualization confirms the model’s strong ability to provide accurate, student-level performance predictions, supporting its practical utility in monitoring physical training programs.

## Discussion

Our study introduces innovative machine learning and deep learning methods in the context of physical fitness assessment for primary school students over the past five years. In comparison to conventional methods, our approach incorporates advanced techniques such as Long Short-Term Memory (LSTM) networks, providing a more sophisticated and accurate analysis of students’ physical health. The key distinction lies in the limited consideration of LSTM in previous physical fitness prediction studies. While various machine learning and deep learning algorithms have been explored, LSTM’s unique ability to handle time-series data and capture sequential dependencies has been underutilized.

Our establishment of a comprehensive evaluation index system for primary school students, utilizing quantitative analysis and the Analytic Hierarchy Process (AHP) for weighting, represents a systematic and data-driven approach to assessing various facets of students’ physical health. The integration of LSTM into our performance prediction model, alongside CNN, marks a significant advancement toward achieving more nuanced and accurate predictions. The advantages of our proposed methods become evident when considering the limitations of traditional physical fitness assessment approaches. The conventional methods often struggle with handling time-dependent factors, leading to less accurate predictions. In contrast, our models, especially those incorporating LSTM, excel in capturing intricate temporal dependencies, offering a more reliable representation of physical fitness progress.

**Fig. 6:**
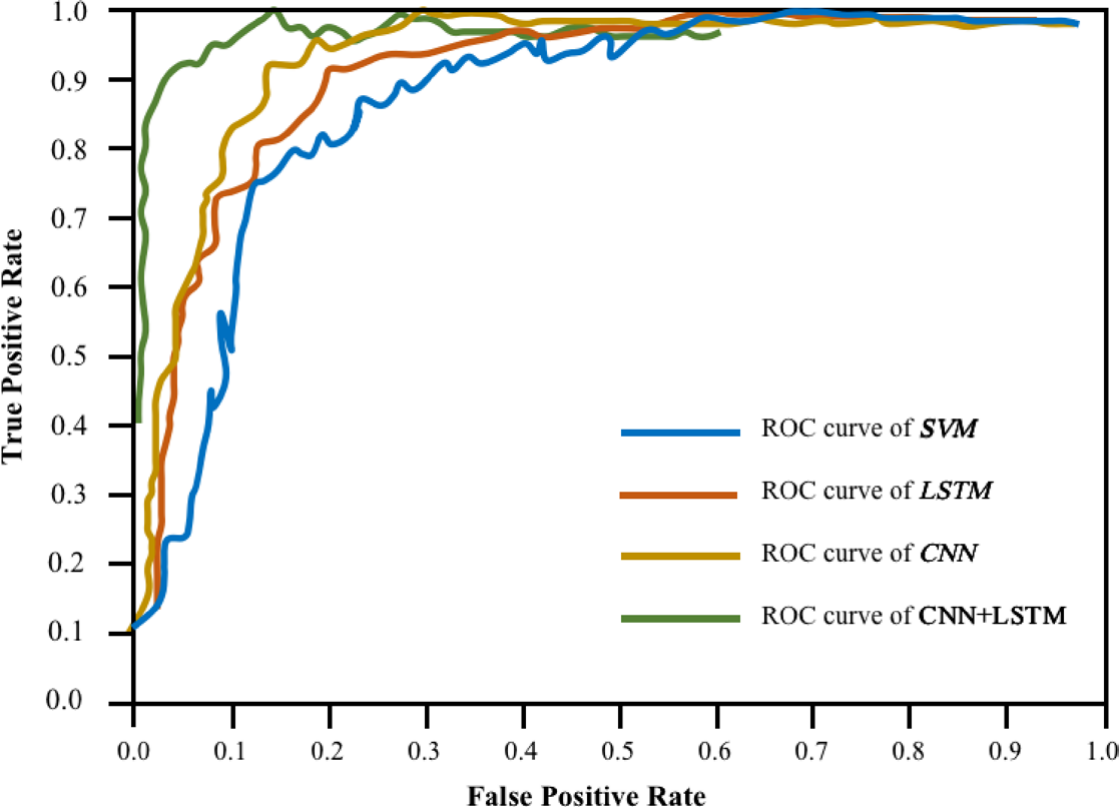
Receiver operator characteristics of the developed four models. SVM, LSTM, CNN and CNN+LSTM.

The Receiver Operator Characteristic (ROC) curve serves as a visual representation to assess the classification models’ detection capabilities, plotting the true-positive against false-positive rates. Models positioned closer to the top-left corner exhibit superior performance compared to those closer to the 45° line. Figure 6 illustrates the ROC curves for SVM, CNN, LSTM, and CNN+LSTM models in predicting students’ fitness test outcomes. The AUC values for the SVM and LSTM models are 0.94 and 0.95, respectively. Correspondingly, the AUC values for the CNN and CNN+LSTM models are 0.96 and 0.97. Upon comparison of AUC values, it is evident that the CNN+LSTM models exhibit comparable performances, as depicted in the Fig. 6.

The progress of artificial intelligence technology and big data has brought new development opportunities for the combination of sports and engineering fields. First of all, the application of artificial intelligence in sports evaluation has obvious advantages. The model designed in this paper can accurately and efficiently analyze physical measurement data, make predictions and calculations, and provide students with personalized teaching suggestions.

This study provides beneficial empirical results for the analysis of physical measurement data and provides a method for the evaluation of individual health status.we analyzed college students’ physical fitness test data to study the patterns and accomplished two tasks: Firstly, visualizing the data to get statistical summary of the data and correlations among different test items; Secondly, predicting students’ test performance using data mining techniques and artificial intelligence. We visualized the data to obtain basic information among different grade and test item get correlation among different test items. We explored 2 state-of-the-art classification algorithms to predict physical fitness test results. We found the proposed CNN+LSTM is the best model in our experiments.

Then, combining the performances of various models in the prediction of physical test items, the CNN-LSTM model proposed in this paper is outstanding in comprehensive performance and performance prediction, and becomes the best choice for physical test performance prediction. In conclusion, our study contributes to the evolution of physical fitness assessment by introducing novel methodologies that address the shortcomings of traditional approaches. The incorporation of advanced algorithms, particularly LSTM, provides a more robust foundation for predicting and understanding students’ physical capabilities. The comprehensive evaluation index system further enhances the precision of our assessments.

Although the primary focus of the current research is on accurate prediction of students’ physical fitness outcomes, these predictions can directly inform personalized pedagogical interventions. Specifically, by analyzing predicted performance trajectories across different physical test items, teachers can identify individual students who may be at risk of declining physical capacity or uneven development in certain fitness dimensions. For example, if the model predicts a gradual decline in a student’s endurance, educators can adjust the intensity or duration of cardiovascular exercises in that student’s training plan. Similarly, predicted weaknesses in muscular strength, flexibility, or coordination can guide targeted exercises to improve those areas. In this way, while the model does not automatically generate training plans, it serves as a data-driven tool to help teachers make informed, personalized decisions, ensuring that physical education interventions are adaptive, precise, and responsive to each student’s needs.

Finally, on this basis, we can further explore the combination of more models and technologies in the future, can further refine our understanding of the factors influencing physical health. Additionally, the integration of personalized training plans and informed curriculum development based on predictive models opens avenues for enhancing the effectiveness of physical education programs in schools. Encourage multi-party cooperation, apply cutting-edge algorithms to realize the deep integration of sports and artificial intelligence, promote the innovation and development in the field of sports, improve the level of physical education, and make greater contributions to students and social health. As technology continues to advance, incorporating these methodologies into broader health assessments and interventions can contribute significantly to the development of more tailored and effective strategies for promoting physical well-being in students.

## Data Availability

The datasets used and analysed during the current study available from the corresponding author on reasonable request.
